# A Textbook of Surgical Anatomy

**Published:** 1911-05

**Authors:** 


**A Textbook of Surgical Anatomy.** By **William Francis Campbell,** M.D., Long Island College Hospital. Second edition revised. Octavo, 675 pp. with 319 original illustrations. Philadelphia and London; W. B. Saunders & Co. 1911. (Cloth, $5.00 net.)

A second edition of this valuable book has just appeared, attractive in size, printed upon beautiful paper, and richly illustrated by fine plates, made largely from original drawings and dissections. The author says, anatomic facts are dry and only have a living interest when associated with clinical values. In this book, he has made anatomy especially interesting by giving anatomic landmarks, which make possible, successful surgery. The subjects are taken up in six parts and under each section, are a number of different chapters devoted to special fields of work, and the surface bearings and clinical importance of each are discussed from these standpoints.

Part I deals with the head and neck in a very comprehensive manner, and the chapters on cerebral localisation illuminate this subject and makes it of great value to the surgeon, who is contemplating this difficult and complex class of work. The plates and subject matter on the thyroid region are very satisfactory, and must be thoroughly mastered, in order to meet the growing importance of the thyroid gland and the parathyroids. The effects of posture, pressure and injury to the nerves of the extremities are thoroughly considered, and the accompanying drawings are especially worthy of comment.

Chapter twenty deals with the abdomen and its contents and is the finest exposition of applied anatomy and surgery, which it has been our privilege to study. In fact, the whole book is entitled to our fullest indorsement and every surgeon, physician, and medical student should have a volume upon his table and consult it freely, in order to make possible, correct diagnoses and to carry out properly the various lines of intervention. H. E. H.

